# A quantitative shotgun proteomics analysis of germinated rice embryos and coleoptiles under low-temperature conditions

**DOI:** 10.1186/s12953-015-0082-5

**Published:** 2015-11-18

**Authors:** Joohyun Lee, Wondo Lee, Soon-Wook Kwon

**Affiliations:** Department of Applied Bioscience, Konkuk, University, Seoul, 143-701 Republic of Korea; Department of Plant Bioscience, Pusan National University, Milyang, 627-706 Republic of Korea

## Abstract

**Background:**

At low temperatures, rice grains have a reduced germination rate and grow more slowly, which delays the emergence of rice seedlings from the paddy water surface and significantly increases seedling mortality. In this study, we conducted a shotgun proteomics analysis of geminated embryos and coleoptiles to compare the proteome expression pattern between the low-temperature resistant variety, Tong 88-7, and the low-temperature susceptible variety, Milyang 23.

**Results:**

In a shotgun proteomics analysis of low-temperature resistant and susceptible embryos and coleoptiles in both cold and control temperatures, we discovered a total of 2626 non-redundant proteins, with a 0.01 false discovery rate. A comparison of protein expression patterns between resistant and susceptible embryos and coleoptiles under low-temperature and normal conditions revealed that 85 proteins and 196 proteins were expressed by the resistant and susceptible strains, respectively, in response to low temperature. Among them, 12 proteins overlapped. Proteins involved in stress responses, metabolism, and gene expression were expressed in both strains.

**Conclusions:**

Similar molecular functions of the response were detected, suggesting that the resistant and susceptible strain have a similar proteome response to cold temperatures. The resistance of Tong 88-7 to cold-water germination may result from the efficiency of these proteins, rather than activation of additional or different molecular processes. A comparison of protein expression between the resistant and susceptible strains’ responses revealed that the more successful low-temperature germination of Tong 88-7 was associated with gibberellin signaling, protein trafficking, and the ABA-mediated stress response.

**Electronic supplementary material:**

The online version of this article (doi:10.1186/s12953-015-0082-5) contains supplementary material, which is available to authorized users.

## Background

Rice (*Oryza sativa* L.) is an important crop because it is a staple food for more than 3 billion people in Asia, and it is a model plant for genomics because of its small genome size. Rice is cultivated worldwide in tropical, subtropical, and temperate regions. Throughout the entire cultivation period, temperatures lower than the optimum temperature for each growth stage can cause serious loss of yield. The major system for cultivating rice, especially in Korea, is to transplant rice seedlings into water-filled rice fields. Rice seedlings are cultivated in a greenhouse or in plastic mulched tunnel beds; therefore, low temperatures during the germination and seedling stage do not cause serious problems. Because this rice cultivating system requires a lot of labor, direct seeding is being considered to reduce labor cost [15]. In addition to competition from weeds, possible damage caused by low temperatures is another barrier to direct sowing in paddy rice field [24]. Slow and irregular germination in low-temperature water results in irregular rice emergence and low population density

Germination is mainly affected by two environmental factors, temperature and moisture. The germination process begins when the seed breaks dormancy and absorbs water. This is phase 1 or the imbibition stage. Phase 2 is the activation stage, where various germination-related enzymes are activated, leading to metabolic modifications. Phase 3 is the set of post-germination growth stages. After the activation stage, the coleoptiles emerges from the embryo, but low water temperature can delay the activation stage or increase the time required [27, 36]. Thus, resistance to cold temperatures during germination is important for fast establishment and uniform crop stand [17]. The deleterious phenotypes caused by low temperatures are reduced germination rate and slow growth, which delay emergence of rice seedlings from the paddy water surface, resulting in significantly elevated seedling mortality [18].

The genic region affecting low-temperature germination and seedling vigor was found in a quantitative trait loci (QTL) analysis [11] using backcrossed inbred lines developed from the cross between temperate *japonica* varieties. Map-based cloningrevealed this region, qLTG-3-1, encoded a protein of unknown function [30]. The expression patterns of the qLTG-3-1 gene imply that cross-talk between various signaling pathways and a wide range of metabolic alterations are associated with low-temperature germination ability, affecting defense-related genes, programed cell death, and phytoalexin biosynthesis [10].

Using proteomic analysis, the set of proteins encoded by the genome can be monitored, and the molecular status of a given cell type can be revealed. Based on advances in mass spectrometry, particularly multidimensional protein identification technology (MudPIT), a shotgun proteomic approach was developed for large-scale, high-throughput protein identification [33], in which the comparative analyses of protein expression can be conducted with the label-free method of spectral counting (spectral count, SpC). SpC is linearly correlated with protein abundance over a dynamic range of two orders of magnitude and provides estimates of the relative protein levels between samples, comparable to estimates derived by radiolabeled quantification [8, 20].

Most proteomics studies of cold tolerance in rice used leaf tissue from seedlings or anthers [7, 14, 16, 34]. In this study, we conducted a shotgun proteomics analysis of germinated embryos in order to compare the protein expression patterns between a low-temperature resistant variety, Tong 88-7, and a low-temperature susceptible variety, Milyang 23.

## Results

### Response to low-temperature germination

**Fig. 1:**
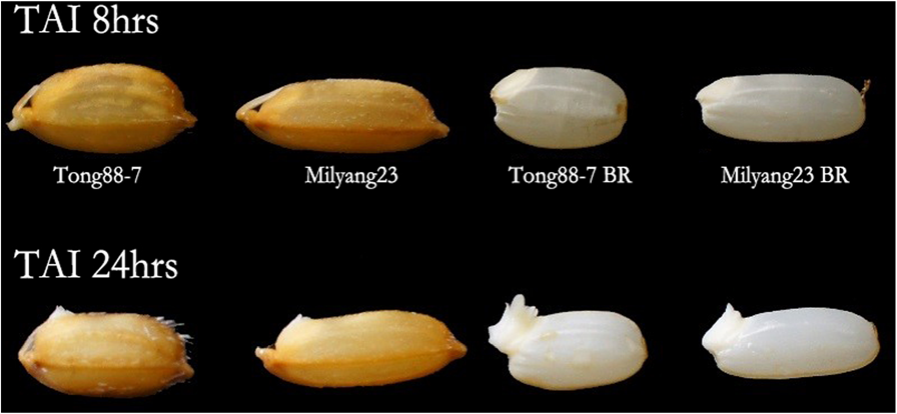
Germinating rough rice and brown rice of Tong 88-7 and Milyang 23 under normal conditions. TAI: time after imbibition (A) Tong 88-7 and (B) Milyang 23

**Fig. 2:**
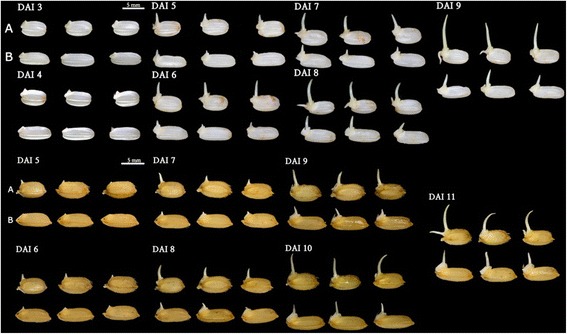
Rough rice from Milyang 23 and Tong 88-7 germinated in cold water (13 °C) for 11 days. (A) Tong 88-7 and (B) Milyang 23

**Fig. 3:**
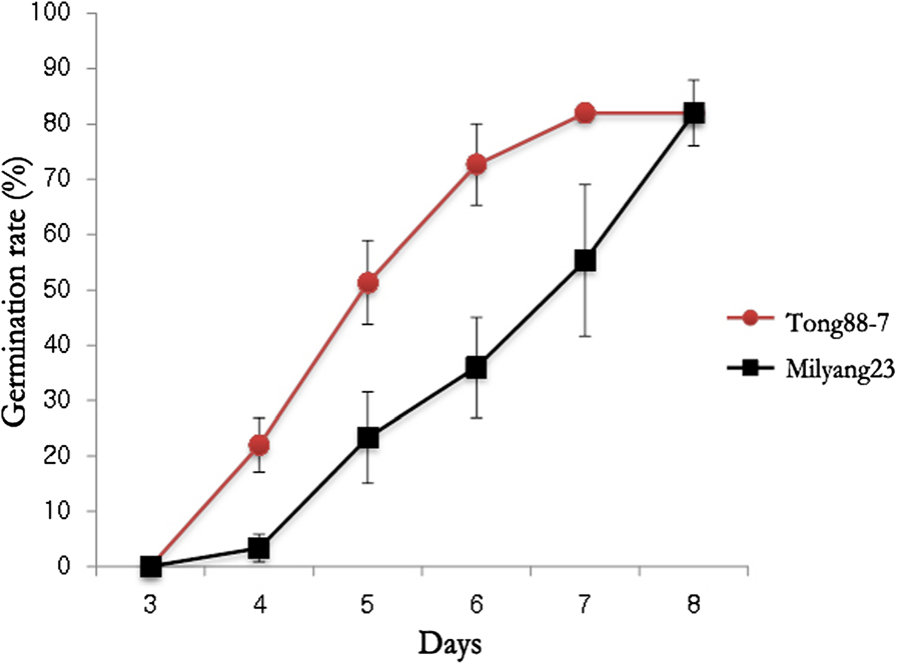
Germination rate of the two varieties, Tong 88-7 and Milyang 23, at 13 °C

**Fig. 4:**
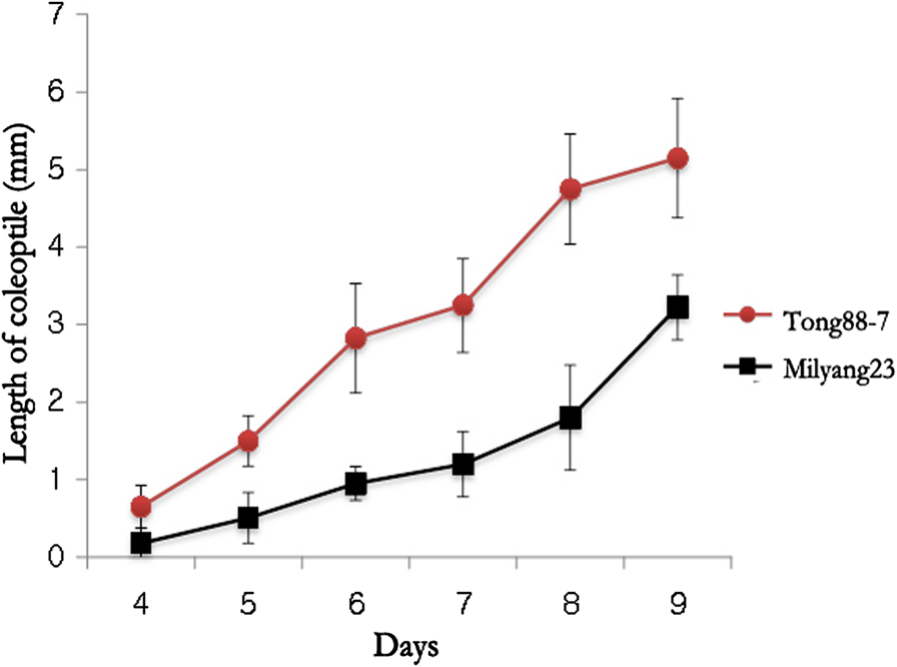
Length of coleoptile of Milyang 23 and Tong 88-7 incubated in cold water (13 °C)

Both Tong 88-7 (the cold-resistant variety) and Milyang 23 (the cold-susceptible variety) germinated under normal conditions, in which water-soaked seeds were incubated at 30 °C in the dark. Both varieties germinated in 24 h from both rough rice and brown rice (Fig. 1), confirming that the seeds used in these experiments were not dormant. The seeds of both varieties were then germinated at 13 °C, and the phenotypic differences were monitored from 1 DAI (days after imbibition). For both the rough and brown rice of both varieties, the emergence of the coleoptile was delayed. A notable coleoptile could be seen at 4 DAI in brown rice and 5 DAI in rough rice (Figs. 2 and 3). In addition, we detected a phenotypically different response between the resistant and susceptible varieties in both rough rice and brown rice, suggesting that the hull does not affect the response to low temperature. Figures 3 and 4 show the comparison of the germination rate and the growth rate of coleoptiles in brown rice. The delay of the germination rate was severe in Milyang 23, as compared to that of Tong 88-7. However, at 8 DAI, both varieties had a ~90 % germination rate (Fig. 3). In addition, after the coleoptile emerged in Tong 88-7, the elongation of the coleoptile could be detected, whereas in Milyang 23, the growth of the coleoptile was retarded (Fig. 4).

### The proteins identified in germinated rice embryo and coleoptile

Because the germination rate and coleoptile growth differed between Tong 88-7 and Milyang 23 under cold conditions, we focused on the proteome expression patterns of the germinated embryos and the coleoptiles. As previously described, the difference of the growth of the coleoptile between Tong 88-7 and Milyang 23 began at 5DAI (Fig. 2) where the morphology of the embryo and coleoptile was similar to the morphology of the embryo and coleoptile under the normal condition at 1DAI. To synchronize the developmental stage used in the proteomic analysis, the germinated embryo and coleoptile under normal conditions were excised at 1 DAI, and the germinated embryo and coleoptile in low temperatures were excised at 5 DAI. All the excised embryos had a ~1 mm coleoptile. With the shotgun proteomic analysis, including 4 sampling points (including 2 controls and 2 treatments) and three replications, a total of 2626 non-redundant proteins were identified, with a 0.01 false discovery rate (Additional file 1: Table S1). However, the number of identified proteins varied from 1169 to 1824 in each sample, most likely because of the analytical incompleteness phenomenon, in which any single analytical run may only identify a fraction of the relevant peptides in a complicated peptide mixture [32]. Because of this, it is hard to judge whether the variation resulted from the treatment; however, the variation was smaller between replications than between different treatments (Additional file 2: Table S2), implying the analysis was conducted reproducibly.

We analyzed the physiochemical properties of the 2626 non-redundant proteins. First, we compared the distributions of pI values and molecular weights (MWs) of the proteins relative to those of all proteins encoded by the rice genome (TIGR Rice Pseudomolecule Database, Release V7.0) (Additional file 3: Figure S1.). The pI values of the identified proteins ranged from 3.7 (LOC_Os03g46600.1) to 12.5 (LOC_Os02g56014.1), a distribution similar to that of the proteins encoded by the entire genome, although there were fewer identified proteins with a pI above pH 8. This could have resulted from the experimental difficulty of solubilizing basic proteins during our protein extraction. Notwithstanding, basic proteins (over pH 7.0) were identified more than 45.8 % of the time, suggesting that our experiment was unbiased. In addition, the MW of the identified proteins ranged from 5.2 kDa (LOC_Os04g02670.1) to 486 kDa (LOC_Os09g07300.1), a distribution similar to that of the proteins encoded by the entire genome, although there were fewer proteins that were smaller than 20 kDa. This is possibly due to the fact that smaller proteins produce fewer peptides during trypsin digestion, meaning that they will have a lower chance of being detected.

### Differentially expressed proteins during low-temperature germination

The relative abundances of the identified proteins were quantified by spectral counting, a label-free method (Materials and Methods). Not all of the 2626 proteins were reproducibly identified in all experiments. Thus, only proteins identified in all three biological replicates with at least two SpCs in each replicate were used in the comparative analysis. A total of 1510 proteins were globally normalized (normalized spectral count, NSpC) (Additional file 4: Table S3) and subjected to a t-test using the logarithmically transformed NSpC values (the natural log (ln) of NSpC). For calculating NSpC, 0.1 was added to the 0 value of SC. The average coefficient of determination (R^2^) between NSpCs for the biological replicates was 0.78–0.95 (Additional file 5: Figure S2), suggesting that the measurements were reproducible. The proteome expression under normal conditions served as the control. The resistance response was identified using a t-test comparing the proteome expression patterns of Tong 88-7 in the low-temperature condition to that of Tong 88-7 in the normal condition. By contrast, the susceptible strain’s response was identified using the expression patterns of Milyang 23. Among proteins identified using the t-test with α = 0.05, the proteins with a greater than 2-fold expression difference were categorized as being differentially expressed between conditions.

**Fig. 5:**
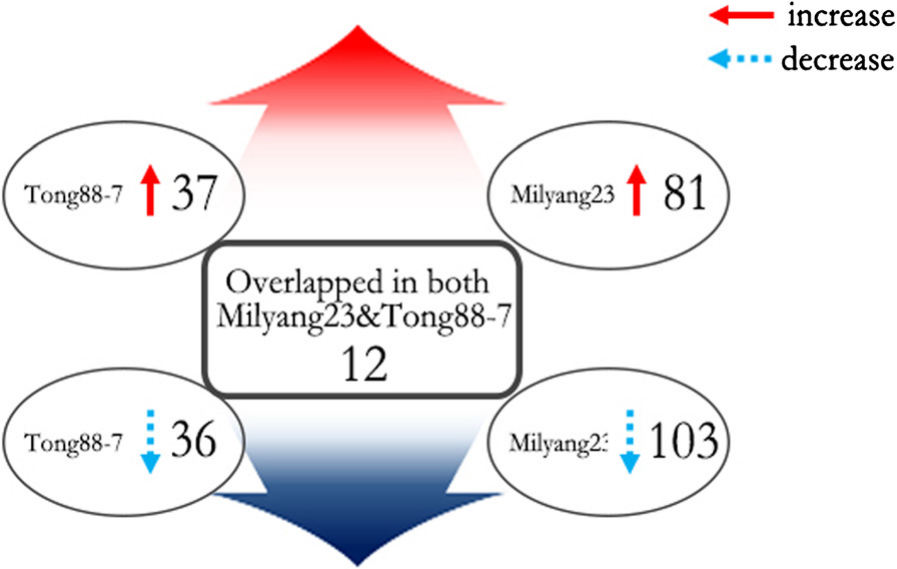
Summary of the differentially expressed proteins in the Tong88-7 and Milyang 23

The differentially expressed proteins detected in both plant varieties were categorized into three groups: the proteins detected only in the response of Tong 88-7, proteins detected only in the response of Milyang 23, and proteins detected in the response of both varieties. The list of proteins and their expression ratio of all of the differentially expressed proteins were represented in Additional file 6: Table S4. The number of the differentially expressed proteins is summarized in Fig. 5 and the list of top 10 differentially expressed proteins and several noticeable proteins for both varieties were represented in Table 1. In the cold-tolerance response of Tong 88-7, 85 proteins were differentially expressed; 73 proteins were detected only in the response of Tong 88-7, whereas 12 proteins were also detected in the cold-susceptible strain Milyang 23. Among the 73 specifically expressed proteins, 37 had increased expression and 36 had decreased expression. In the cold-susceptible Milyang 23, 196 proteins were differentially expressed and 184 proteins were detected only in the response of Milyang 23. Of the total, 81 had increased expression and 103 had decreased expression. The highly expressed protein in tolerant response was ATP synthase (LOC_Os 01 g 10720.1) and in the susceptible response was S-adenosylmethionine synthetase (LOC_Os01g22010.2) which is the enzyme that catalyzes the formation of S-adenosylmethionine (AdoMet) from methionine and ATP [9]. Using the Gene Ontology (GO) database, we categorized the proteins into specific functional categories. In the susceptible plant’s response, we observed an enrichment in 5 GO categories related to biological processes (Table 2), 1 category related to molecular functions, and 32 categories related to cellular components (Additional file 4: Table S3). In the resistant strain’s response, we observed an enrichment in 8 GO categories related to biological processes (Table 2), 1 category related to molecular functions, and 22 categories related to cellular components (Additional file 7: Table S5). In the resistant response specific GO term of transport, the decrease of 3 aquaporin proteins (LOC_Os07g26690.2, LOC_Os03g05290.1, and LOC_Os04g47220.1) was detected in the cold treated resistant variety. Aquaporin in plant is known for the possible role in chilling stress in maize root [1]. The opposite expression pattern in our experiment of the cold resistant variety is not clearly explained based on our proteomic results. Unlike aquaporin, outer mitochondrial membrane porin (LOC_Os03g04460.1) which is known for allowing diffusion of small hydrophilic molecules was increased. In the previous report for rice, its transcript level was the highest during the first days following the germination, suggesting the possible role in development of coleoptiles in rice germination [25].

**Fig. 6:**
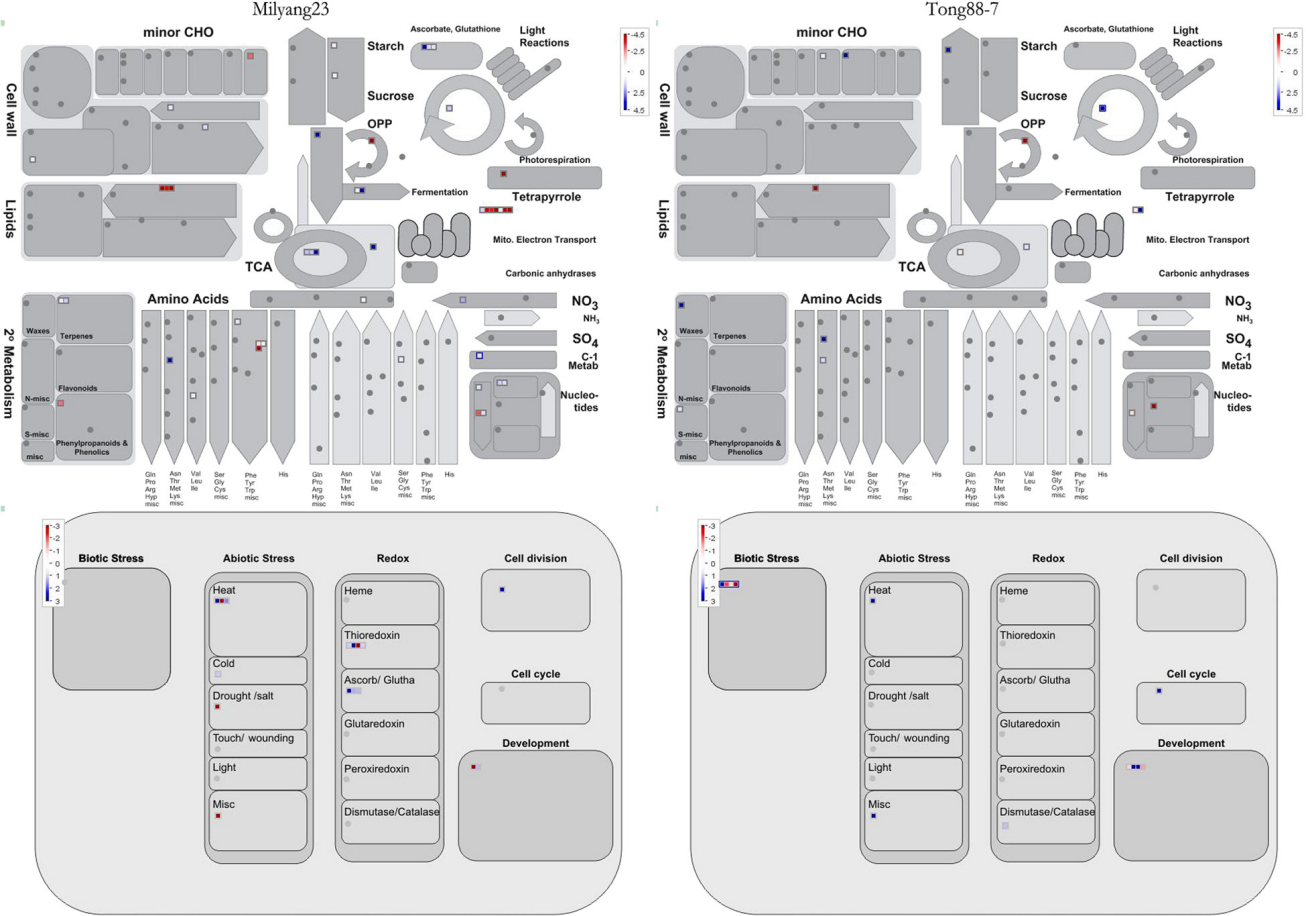
The increased or decreased genes under the cold treatment in the Tong 88-7 and Milyang 23 encoding enzymes involved in metabolism and cellular response grouped in the functional bins according to MapMAn. Red indicates down-regulated, blue up-regulated genes

**Table 1:** The list of top 10 differentially expressed proteins and several noticeable proteins in resistant and susceptible responses

Accession	Description	Expression ratio (cold treatment/control)
Tong88-7		
LOC_Os01g49190.1	ATP synthase, putative, expressed	1770.556
LOC_Os07g10720.1	40S ribosomal protein S15a, putative, expressed	157.1791
LOC_Os08g41810.1	ribosomal protein L22, putative, expressed	107.4843
LOC_Os03g46490.1	40S ribosomal protein S21, putative, expressed	92.12076
LOC_Os09g12660.1	glucose-1-phosphate adenylyltransferase large subunit, chloroplast precursor, putative, expressed	71.75172
LOC_Os08g25590.2	UPF0041 domain containing protein, putative, expressed	0.012783
LOC_Os03g22460.2	expressed protein	0.011671
LOC_Os03g05290.1	aquaporin protein, putative, expressed	0.008718
LOC_Os07g02880.1	DUF538 domain containing protein, putative, expressed	0.008399
LOC_Os02g27760.1	40S ribosomal protein S15a, putative, expressed	0.005065
LOC_Os07g26690.2	aquaporin protein, putative, expressed	0.450865
LOC_Os03g05290.1	aquaporin protein, putative, expressed	0.008718
LOC_Os04g47220.1	aquaporin protein, putative, expressed	0.297092
LOC_Os03g04460.1	outer mitochondrial membrane porin, putative, expressed	59.73071
LOC_Os01g06560.1	transcription factor HBP-1b, putative, expressed	41.88637
LOC_Os02g35940.1	gibberellin receptor GID1L2, putative, expressed	32.93806
LOC_Os07g07000.1	syntaxin, putative, expressed	6.51711
LOC_Os07g26700.1	UPF0041 domain containing protein, putative, expressed	41.72113
LOC_Os03g08810.1	expressed protein	6.349671
LOC_Os01g23610.1	dihydrolipoyl dehydrogenase, putative, expressed	0.46724
LOC_Os02g03260.3	3-isopropylmalate dehydratase large subunit 2, putative, expressed	2.382451
LOC_Os01g65830.1	acyl-desaturase, chloroplast precursor, putative, expressed	0.019233
Milyang23		
LOC_Os01g22010.2	S-adenosylmethionine synthetase, putative, expressed	359.1924
LOC_Os08g13690.1	60S ribosomal protein L7, putative, expressed	278.6428
LOC_Os02g49720.6	aldehyde dehydrogenase, putative, expressed	249.0047
LOC_Os01g52500.3	NADP-dependent malic enzyme, putative, expressed	206.6945
LOC_Os05g06750.1	dihydrolipoyl dehydrogenase, mitochondrial precursor, putative, expressed	155.0024
LOC_Os01g32800.2	proteasome subunit, putative, expressed	0.014422
LOC_Os09g21110.1	leucyl-tRNA synthetase, cytoplasmic, putative, expressed	0.013035
LOC_Os08g02400.1	40S ribosomal protein S13, putative, expressed	0.009922
LOC_Os03g13800.1	ribosomal protein L7Ae, putative, expressed	0.009743
LOC_Os01g23620.1	miro, putative, expressed	0.007213
LOC_Os02g07490.1	glyceraldehyde-3-phosphate dehydrogenase, putative, expressed	59.9627
LOC_Os12g08170.1	2-oxo acid dehydrogenases acyltransferase domain containing protein, expressed	3.578667
LOC_Os01g22520.1	dihydrolipoyl dehydrogenase 1, mitochondrial precursor, putative, expressed	3.457981
LOC_Os03g22950.1	acyl carrier protein, putative, expressed	0.041124
LOC_Os09g36860.1	acyl carrier protein, putative, expressed	0.059347
LOC_Os08g06550.1	acyl CoA binding protein, putative, expressed	0.040972
LOC_Os04g52130.1	coproporphyrinogen III oxidase, chloroplast precursor, putative, expressed	0.023115

**Table 2:** The enriched GO terms concerning biological processes for the differentially expressed proteins in the resistant response of Tong88-7 and the susceptible response of Milyang 23

	GO term	Description	Number in input list	Number in rice genome	*P*-value	FDR
Tong 88-7						
	GO:0044249	cellular biosynthetic process	9	890	0.00024	0.007
	GO:0034645	cellular macromolecule biosynthetic process	9	890	0.00024	0.007
	GO:0009059	macromolecule biosynthetic process	9	890	0.00024	0.007
	GO:0006412	translation	9	890	0.00024	0.007
	GO:0010467	gene expression	9	1076	0.00094	0.022
	GO:0006810	transport	16	3278	0.0035	0.05
	GO:0051234	establishment of localization	16	3278	0.0035	0.05
	GO:0051179	localization	16	3278	0.0035	0.05
Milyang 23						
	GO:0044249	cellular biosynthetic process	18	890	2.80E-06	0.00014
	GO:0034645	cellular macromolecule biosynthetic process	18	890	2.80E-06	0.00014
	GO:0010467	gene expression	20	1076	2.60E-06	0.00014
	GO:0009059	macromolecule biosynthetic process	18	890	2.80E-06	0.00014
	GO:0006412	translation	18	890	2.80E-06	0.00014

Some similarities in the responses were apparent. In both responses, a variety of cellular components were active, but only one GO category related to a molecular function was active: structural molecular activity. For GO terms regarding biological processes, both responses had an enrichment in cellular biosynthetic processes, cellular macromolecule biosynthetic processes, macromolecule biosynthetic processes, and gene expression. This result reflects the molecular status of the tissue sample, the embryo, where cell divisions are occurring dynamically and the cold treatment is affecting cell division and new cell development in both resistant and susceptible plants.

### Proteins specifically expressed in the resistant plant’s response

Among the 12 proteins that overlapped in the both responses, the expression patterns of ten were similar, but two of the ribosomal proteins (LOC_Os07g47780.1 and LOC_Os08g41810.1) had elevated expression in the resistant variety’s response and decreased expression in the susceptible variety’s response. However, changes in the expression of several other ribosomal proteins were detected in both the resistant and susceptible strains’ responses, but unexpectedly, there was no consistency in the expression patterns between the responses. Expression of some ribosomal proteins was elevated, whereas expression of others was reduced, following cold treatment of both resistant and susceptible plants. Based on the general molecular functions of the 73 proteins were specifically expressed in the resistant strain, among the 73 proteins, the function of some proteins were overlapped with those of the proteins specifically expressed in the susceptible strain; such as stress response, metabolism, and gene expression. By eliminating the proteins that functionally overlapped with proteins expressed in the susceptible variety’s response, we identified several proteins with elevated expression only in the resistant strain’s response: the transcription factor HBP-1b (LOC_Os01g06560.1), gibberellin receptor GID1L2 (LOC_Os02g35940.1), syntaxin (LOC_Os07g07000.1), and two proteins with unknown functions, UPF0041 domain-containing protein (LOC_Os07g26700.1) and expressed protein LOC_Os03g08810.1, were identified. These proteins represent candidate factors involved in the resistance of Tong 88-7 to cold germination.

## Discussion

### Comparing the proteome response between resistant and susceptible plants

In this proteome analysis, proteome expression changes in resistant and susceptible response were monitored and total of 256 differentially expressed proteins were detected. Among them, only 12 proteins were detected in both resistant and susceptible responses. According to the function of the specifically detected proteins in both responses, there were overlapped functional categories in both resistant and susceptible responses such as stress response, cellular macromolecule biosynthesis, and translation. This result suggests that certain proportion of resistance in Tong 88-7 to cold water may be resulted from the efficiency of these stress-associated proteins. Supporting this, the stress defense proteins that were present in both responses were expressed more in the resistant plant’s response than in the susceptible plant’s response. For instance, two peroxidase precursors (LOC_Os04g59160.1 and LOC_Os07g48010.1) that are involved in the removal of H_2_O_2_ had increased levels in both responses; however, in the resistant strain’s response, the levels of LOC_Os04g59160.1 had increased 9.56-fold, and those of LOC_Os07g48010.1 had increased 62.03-fold, whereas in the susceptible strain’s response, the expression of those proteins had only increased 3.14- and 13.96-fold, respectively. In addition, chaperone protein dnaJ (LOC_Os03g44620.2), which is associated with protein folding, unfolding, and assembly under both normal and stress conditions [31], had 56.76-fold expression in the resistant strain’s response, whereas it had only increased 13.96-fold in the susceptible plant’s response.

Rice seed germination involves metabolic changes that mobilize reserves, synthesize new proteins, and regenerate organelles. The activities of enzymes related to starch biosynthesis are also elevated in the embryo. An increase in an enzyme involved in glycolysis, the TCA cycle, starch synthase, and a starch branching enzyme involved in amylopectin biosynthesis, has been detected in germinated rice seed [13]. A Mapman analysis [29] revealed changes in the expression of a few proteins involved in glycolysis and the TCA cycle in both responses (Fig. 6); however, the expression patterns of these proteins were not concordant. In the susceptible response, levels of a protein involved in glycolysis (LOC_OS02g07490.1: glyceraldehyde-3-phosphate dehydrogenase) were reduced, whereas levels of proteins involved in the TCA cycle (LOC_OS12g08170.1: 2-oxo acid dehydrogenases acyltransferase domain-containing protein; LOC_OS01g22520.1: dihydrolipoyl dehydrogenase 1, mitochondrial precursor; and LOC_OS05g06750.1: dihydrolipoyl dehydrogenase, mitochondrial precursor) were elevated. In addition, in the resistant variety’s response, no expression changes were detected for proteins involved in glycolysis, and the changes in the expression pattern of the two proteins in the TCA cycle (LOC_OS01g23610.1: dihydrolipoyl dehydrogenase and LOC_OS02g03260.3: 3-isopropylmalate dehydratase large subunit) were in opposite directions. Moreover, no expression changes were detected for proteins related to starch synthesis. These results imply that the primary starch metabolism was not influenced by cold treatment. This may be due to the fact that the morphology of germinating embryos used in the proteomic analysis did not differ significantly.

During cold stress, damage to membrane lipids and subsequent membrane instability can occur. Plant membrane lipids have a tendency to desaturate in response to temperature stress, a process caused by fatty-acid desaturases [3]. In this study, we detected reduced expression of proteins in fatty-acid synthesis in both responses. In the susceptible plant’s response, three proteins (LOC_OS03g22950.1: acyl carrier protein; LOC_OS09g36860.1: acyl carrier protein; and LOC_OS08g06550.1: acyl CoA binding protein) had decreased expression, whereas in the resistant plant’s response, only one protein (LOC_OS01g65830.1: acyl-desaturase, chloroplast precursor) did. It is not clear how these fatty-acid synthesis proteins affected the response.

In the susceptible plant’s response, expression of coproporphyrinogen III oxidase (LOC_OS04g52130.1), which is involved in the biosynthesis of tetrapyrrole, was reduced by 5-fold, whereas the expression did not change in the resistant variety. Tetrapyrroles are a family of compounds in plants, such as chlorophyll, that contain four pyrrole rings. As regulators of protein activity, tetrapyrroles mediate the cellular response to light, oxygen, and abiotic stress [35], and modified tetrapyrrole biosynthesis in plants prevents wilting during drought stress [23]. Even though we did not detect elevated levels of coproporphyrinogen III oxidase in the resistant variety’s response, the decreased level of this protein in the susceptible variety’s response suggests that it plays a role in the cold stress response.

### Proteins specific to the resistant strain’s response

As in other omics studies of stress responses, we found various pathways related to the stress response in both strains. To find proteins specific to the resistant strain’s response, we compared the proteins that were differentially expressed between strains and found only a small number were in both responses. However, as discussed previously, the known functions of the majority of the proteins were similar, meaning the overall proteome response was similar in both plants. This perhaps results from the genomic backgrounds of the two varieties; Tong 88-7 is a *japonica* strain, whereas Milyang 23 is a Tongil rice from a cross between the *indica* and *japonica* subspecies.

We focused on five resistance response-specific proteins that had a function that could increase the cold resistance of germinating seeds. Gibberellin (GA) is a plant hormone that affects a wide range of processes including plant growth, development, and environmental responses, including seed germination, stem elongation, leaf expansion, pollen maturation, and the induction of flowering [28]. Increasing GA biosynthesis and GA signaling is linked to stress tolerance [6]. GA-related proteins have increased expression during rice seed germination [2]. Because of this, GA could play an important role in the elongation of the coleoptile in cold temperatures. The elevated expression of the GA receptor GID1L2 (LOC_Os02g35940.1) in the resistant strain’s response suggests that GA mediated the response to cold temperature in the germinating embryo.

Expression of one transcription factor, HBP-1b (LOC_Os01g06560.1), was elevated in the resistant strain’s response. HBP-1b is a leucine zipper-type transcription factor [26]. In *Arabidopsis*, the cloned gene for seed dormancy (DOG1) encodes a member of a plant-specific protein family that shares a domain with D class leucine zipper-type transcription factors [4]. In rice, there is no direct evidence for the association of HBP-1b to germination. However, Li et al. [19] reported that the zipper-type transcription factor LOC_Os01g06560 is located inside of a QTL region for seed dormancy, and LOC_Os01g06560 is homologoues with *Arabidopsis* DOG1, with 28 % identity and a low *P*-value. Based on the elevated expression of the transcription factor HBP-1b in the resistant strain’s response, we hypothesize that LOC_Os01g06560 is involved in either seed germination or cold resistance in the germinating embryo.

Expression of syntaxin (LOC_Os07g07000.1) was also elevated. The syntaxin proteins are involved in vesicle sorting, docking, and fusion in the secretory pathway. In plants, syntaxin is involved in general mechanisms of protein trafficking through the secretory pathway [37]. In *Arabidopsis*, syntaxin plays an important role in ABA-mediated stomatal control during drought stress [38].

Two proteins of unknown function, UPF0041 domain-containing protein (LOC_Os07g26700.1) and expressed protein LOC_Os03g08810.1, had increased expression, suggesting a function in seed germination or cold stress. LOC_Os03g08810.1 is classified into the category of embryo development in the GO database, supporting its presence in the rice embryo and its function in germination.

Even though the previously reported functions of the proteins that were specifically expressed in the resistant strain support the association of these proteins to cold resistance in germinating rice, that is only indirect evidence, and further analysis, such as overexpression or knockout experiments, is required.

## Conclusions

In summary, through quantitative shotgun proteomics analysis of low-temperature resistant and susceptible embryos and coleoptiles in both cold and control temperatures, we identified a total of 2626 non-redundant proteins, which is one of the large scale rice protein identification. A comparison of protein expression patterns between resistant and susceptible embryos and coleoptiles under low-temperature and normal conditions revealed that proteins involved in stress responses, metabolism, and gene expression were expressed in both strains and there were similar molecular functions of the both response detected, suggesting that certain proportion of the resistance of Tong 88-7 to cold-water germination may result from the efficiency of these proteins, rather than activation of additional or different molecular processes. A comparison of protein expression between the resistant and susceptible strains’ responses revealed that the more successful low-temperature germination of Tong 88-7 was associated with gibberellin signaling, protein trafficking, and the ABA-mediated stress response.

## Methods

### Plant materials and the evaluation of low-temperature germination

Tong 88-7, a temperate *japonica* variety that was developed in northeast China, is resistant to low temperatures [21]. Milyang 23 is a Tongil rice variety. Tongil rice is similar to the *indica* variety and is susceptible to low temperatures [5]. For both the resistant and susceptible rice seeds, 50 brown rice grains were placed on filter paper in a petri dish. On the first day, 15 ml of 13 °C distilled water was added. The petri dishes were incubated at 13 °C in an incubator. For the control, brown rice from Tong 88-7 and Milyang 23 was incubated at 28 °C. Three biological replicates were conducted.

### Protein extraction

After the coleoptile emerged from the germinated brown rice, the embryo and its coleoptile were excised. To synchronize the developmental stages, the germinated brown rice embryos in the control treatment were collected at 1 DAI, whereas in the low-temperature condition, the embryos were collected at 5 DAI. To extract enough protein for analysis, more than ten embryos from the same petri dish were pooled. Harvested embryos were ground in liquid nitrogen using a mortar and pestle. Protein was extracted from the resultant powder with extraction buffer (100 mM Tris-HCl, pH 8.5, 8 M urea, 5 mM DTT, and 1 % LDS). The suspension was incubated at room temperature for 30 min, followed by centrifugation at 14,000 × g for 15 min. The supernatant was retained and filtered through 0.45 μm membrane filters (Millipore, Billerica, MA, USA). The protein concentration was assayed using the 2D-Protein Quant Kit (GE Healthcare, Piscataway, NJ, USA).

### 1D LDS-PAGE and in-gel digestion by trypsin

A total of 50 μg of protein was loaded into a 4–12 % Bis-Tris acrylamide gradient gel (Invitrogen, Carlsbad, CA, USA). Proteins were stained using the Colloidal Blue Staining Kit (Invitrogen). For each sample, each lane containing separated proteins was sliced into seven pieces. Each piece was additionally cut into a smaller sized cube (approximate size, 1 mm^3^) and then put into an Eppendorf tube (Hamburg, Germany). Sliced gels were destained with destaining buffer (50 mM NH_4_HCO_3_, pH 7.8, in 50 % acetonitrile), dehydrated in a SpeedVac (Thermo Fisher Scientific, San Jose, CA, USA), reduced at 56 °C for 45 min with reduction buffer (10 mM DTT in 25 mM NH_4_HCO_3_), and alkylated for 30 min at room temperature in the dark with alkylation buffer (55 mM iodoacetamide in 25 mM NH_4_HCO_3_). The gel was completely dried in a SpeedVac and mixed with digestion buffer (12.5 ng/μl trypsin in 50 mM NH_4_HCO_3_), and incubated at 36 °C overnight. Peptides were harvested from the gel using harvest buffer (5 % formic acid in 50 % acetonitrile), vortexed, spun down, and stored at room temperature for 20 min. Combined supernatants were dried in a SpeedVac. The samples were desalted using Pierce C18 spin columns (Thermo Scientific, Rockford, IL, USA), and each sample was analyzed with LC MS/MS.

### LC MS/MS analysis with Q exactive HF

A nanoflow HPLC instrument (Easy nLC, Thermo Fisher Scientific) coupled online to a Q Exactive mass spectrometry unit (Thermo Fisher Scientific, Bremen, Germany) was used. Analytical columns (12 cm, 75 μm inner diameter) were packed in-house with Alltima C18-AQ 5 μm resin. Reversed-phase chromatography was performed with a binary buffer system consisting of 0.1 % formic acid (buffer A) and acetonitrile in 0.1 % formic acid (buffer B). The sample was separated with a linear gradient of 3–60 % buffer B at a flow rate of 250 nl/min. The total run time for a LC MS/MS was 110 min. MS data were acquired using a data-dependent top8 method, which dynamically chose the most abundant precursor ions from the survey scan (300–2000 Da) for higher-energy collisional dissociation (HCD) fragmentation. The dynamic exclusion duration was 60 s, and the isolation window of precursors was performed with 4. Survey scans were acquired at a resolution of 70,000 at m/z 200, and the resolution for the HCD spectra was set to 17,500 at m/z 200.

### Protein identification and comparative analysis of relative protein abundances

The MS/MS spectra from the seven sliced gels for each sample were merged and then analyzed by Proteome Discoverer (version 1.3) software. The protein database used was the TIGR Rice Pseudomolecule Database, Release V7.0 (http://rice.plantbiology.msu.edu/annotation_pseudo_current.shtml), with the precursor and fragment mass tolerances set to 10 ppm and 0.8 Da, respectively, and with up to two missed cleavages allowed. Carbamidomethylation of cysteine was set as a fixed modification, and oxidation of methionine was set as a variable modification for database searching. Both peptide and protein identifications were filtered using a 1 % false discovery rate.

The output of Proteome Discoverer (version 1.3) was exported to Microsoft Excel in order to calculate the NSpCs [12, 22, 39]. The NSpC for each protein *k* is given by

$$ {\left(\mathrm{NSpC}\right)}_{\mathrm{k}}\kern0.5em =\kern0.5em \frac{{\left(\frac{\mathrm{SpC}}{\mathrm{L}}\right)}_{\mathrm{k}}}{{\displaystyle {\sum}_{\mathrm{i}=1}^{\mathrm{n}}{\left(\frac{\mathrm{SpC}}{\mathrm{L}}\right)}_{\mathrm{i}}}} $$

where the whole number of MS/MS spectra-matching peptides from protein k (SpC) is divided by the protein’s length (L), then divided by SpC/L for all n proteins in the experiment.

### Bioinformatics analysis of proteomic data

The whole rice protein MW and pI values were calculated by EMBOSS Pepstats, (http://emboss.sourceforge.net/download/) using the EMBOSS Pepstats algorithm. (http://emboss.bioinformatics.nl/cgi-bin/emboss/pepstats) GO annotations of the rice proteins were retrieved from the TIGR Rice Pseudomolecule Database, Release V7.0. GO singular enrichment analyses were performed in agriGO (http://bioinfo.cau.edu.cn/agriGO/analysis.php).

## Additional files

Additional file 1: Table S1. List of total 2626 identified non-redundant proteins and their spectral counts in each sample. (XLS 530 kb) Additional file 2: Table 2. Number of the identified proteins in each treatments and replications. (DOCX 16 kb) Additional file 3: Figure S1. Distributions of pI values and molecular weights (MWs) of the proteins relative to those of all proteins encoded by the rice genome. (JPG 171 kb) Additional file 4: Table S3. Relative expression amount of the 1510 proteins among the treatment and repliactions. (XLS 510 kb) Additional file 5: Figure S2. The average coefficient of determination (R^2^) between NSpCs for the biological replicates. (JPG 584 kb) Additional file 6: Table S4. Differntailly expressed protein in Tong88-7. (XLS 63 kb) Additional file 7: Table S5. Enriched GO terms of the differentially expressed proteins in the resistant response of Tong887 and the susceptible response of Milyang23. (XLSX 70025 kb)
