# Supplementary material for: A quantitative shotgun proteomics analysis of germinated rice embryos and coleoptiles under low-temperature conditions
Source: Proteome Sci. 2015 Nov 18;13:27. doi: 10.1186/s12953-015-0082-5 (PMC4652350; doi:10.1186/s12953-015-0082-5)
Supplement: Additional file 2: Table 2. — Number of the identified proteins in each treatments and replications. (DOCX 16 kb) [file 12953_2015_82_MOESM2_ESM.docx]

Supplementary Table 2. Number of the identified proteins in each treatments and replications

| condition | 1st rep. | 2nd rep. | 3rd rep. | avg | stde |
| --- | --- | --- | --- | --- | --- |
| T_ Con^b^ | 1564.00 | 1609.00 | 1611.00 | 1594.67 | ± 26.58 |
| M_ Con^b^ | 1524.00 | 1539.00 | 1665.00 | 1576.00 | ± 77.44 |
| T_ Treat^a^ | 1750.00 | 1778.00 | 1824.00 | 1784.00 | ± 37.36 |
| M_ Treat^c^ | 1169.00 | 1209.00 | 1320.00 | 1232.67 | ± 78.23 |
| T : T88-7, M :M23, Con : control, Treat : cold-treatment | | | | | |
