# Supplementary figures and images for: A quantitative shotgun proteomics analysis of germinated rice embryos and coleoptiles under low-temperature conditions
Source: Proteome Sci. 2015 Nov 18;13:27. doi: 10.1186/s12953-015-0082-5 (PMC4652350; doi:10.1186/s12953-015-0082-5)

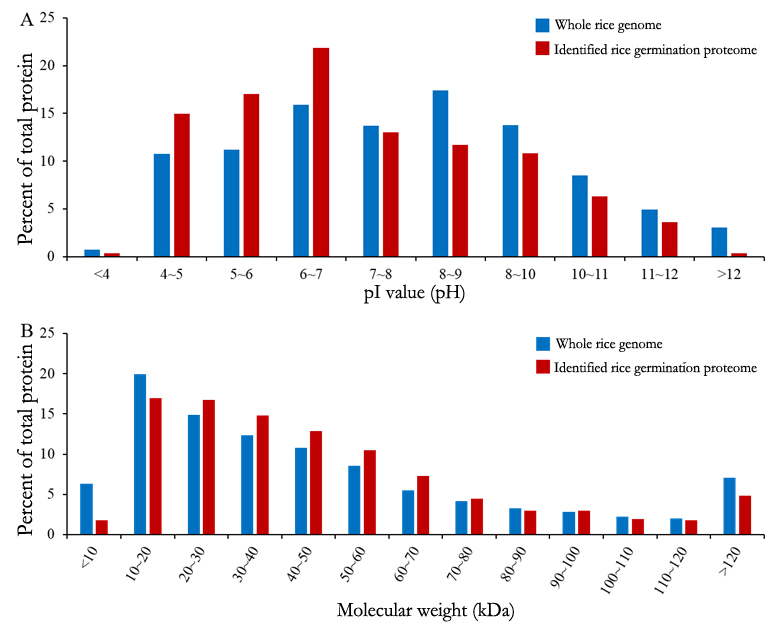

Supplement: Additional file 3: Figure S1. — Distributions of pI values and molecular weights (MWs) of the proteins relative to those of all proteins encoded by the rice genome. (JPG 171 kb) [file 12953_2015_82_MOESM3_ESM.jpg]

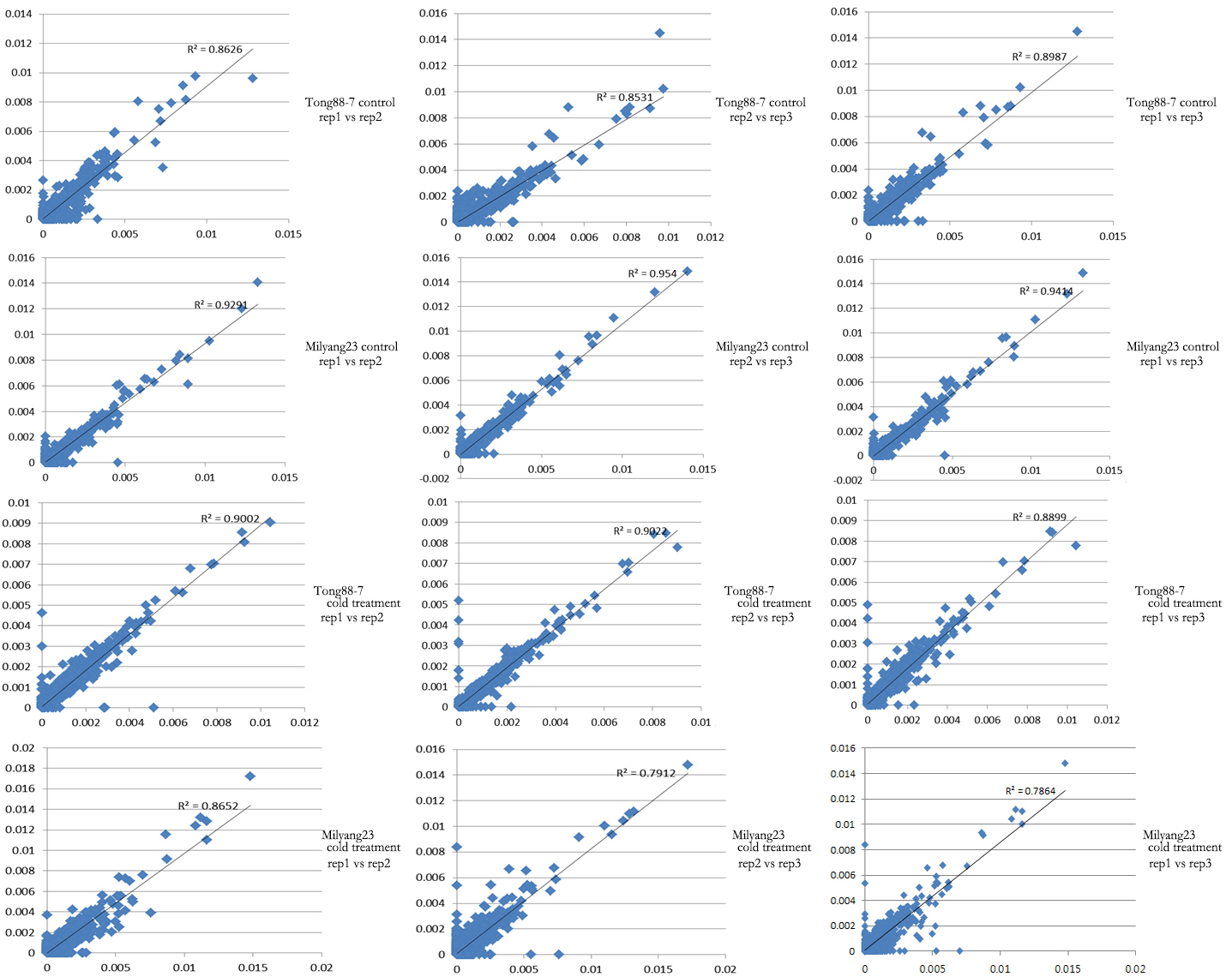

Supplement: Additional file 5: Figure S2. — The average coefficient of determination (R2) between NSpCs for the biological replicates. (JPG 584 kb) [file 12953_2015_82_MOESM5_ESM.jpg]
